# Placental pathology in sudden intrauterine death (SIUD) in SARS-CoV-2-positive oligosymptomatic women

**DOI:** 10.1007/s00404-022-06614-0

**Published:** 2022-06-18

**Authors:** Lars-Christian Horn, Irene Krücken, Grit Gesine Ruth Hiller, Maria Niedermair, Kristina Perac, Corinna Pietsch, Anne Kathrin Höhn

**Affiliations:** 1 Division of Breast Gynecologic and Perinatal Pathology, Institute of Pathology, University Hospital of Leipzig, Liebigstrasse 26, 04103 Leipzig, Germany; 2 Institute of Clinical and Molecular Pathology, City Hospital Wels-Grieskirchen, Grieskirchen, Austria; 3 Institute of Medical Microbiology and Virology, University Hospital of Leipzig, Leipzig, Germany

**Keywords:** Placenta, COVID-19, SARS-CoV-2, Trophoblast, Fibrin deposits, Intrauterine death

## Abstract

**Background:**

Pregnant women are also susceptible to SARS-CoV-2. Although an infection of the placenta may be rare, pregnancy may occasionally be affected by intrauterine failure. The knowledge of placental morphology on sudden intrauterine demise is still limited.

**Methods:**

Fetal and placental tissue of two cases of sudden intrauterine death in the second trimester were analysed morphologically and by immunohistochemistry. One case was evaluated by RT-PCR.

**Results:**

Both mothers were tested positive for the Alpha variant of SARS-CoV-2 but were oligosymptomatic for COVID-19. Unexpected sudden intrauterine death (SIUD) occurred at 15 + 2 and 27 + 3 weeks of gestation. One fetus demonstrated an intrauterine growth restriction. No malformations nor inflammatory changes were observed in either fetus on autopsy. In contrast to the placentas, the fetal tissue was negative for SARS-CoV-2 on immunohistochemical and RT-PCR analyses. Macroscopically, the placentas showed an increased consistency with a white, reticular cutting surface covering about 95% of the whole placenta. Only very focal histiocytic chronic intervillositis was noted histologically. Massive perivillous fibrin deposits with extensive necroses of the villous trophoblast were present in more than 90% of the placental tissue. Immunohistochemical staining was strong and diffusely positive for SARS-CoV-2 in the villous trophoblast and rarely within the villous stromal cells. Placental SARS-CoV-2 infection was confirmed by RT-PCR.

**Conclusion:**

Sudden intrauterine death may occur in mothers who are oligosymptomatic for COVID-19. Acute placental failure is responsible for SIUD, demonstrated by massive perivillous fibrin deposits and extensive necroses of the villous trophoblast with SARS-CoV-2-positivity based on immunohistochemical staining and RT-PCR. Detailed histopathological examination of placental and fetal tissue is mandatory to verify SARS-CoV-2 and to evaluate the pathogenesis and functionality of this disease.

## Introduction

Coronavirus disease 2019 (COVID-19) is caused by SARS-CoV-2 (severe acute respiratory syndrome coronavirus type 2). In addition to the eponymous acute respiratory symptoms [1, 2], many other clinical manifestations of the infection have been reported, including a wide variety of long-term complications summarized as long-COVID [3]. Among others, advanced age and comorbidities such as obesity, diabetes, respiratory and cardiovascular disease, malignant tumors and immunosuppression are considered risk factors for poor prognosis of the infection [4].

There is increasing evidence that pregnant women are also susceptible to SARS-CoV-2 [5–10]. Transplacental vertical transmission of the virus has been reported [5, 6]. However, data about morphologic alteration of the placental tissue and the consequences for the fetus are still limited. We present two cases of pregnant women in the second trimester with subclinical SARS-CoV-2 infection and sudden intrauterine death (SIUD).

## Methods

The placental tissue was examined, and morphologic features were classified according to the published Amsterdam-criteria [11]. At least six blocks of placental parenchyma (three from the periphery and three from the central part) were examined from each case.

Immunohistochemical analyses utilized on-slide positive controls of lung tissue from SARS-CoV-2-positive autopsy controls and tonsillar tissue for CD68. According to the manufacturer’s information, the antibody used is directed against the SARS-CoV-2 nucleocapsid protein with binding specifity at AA 1–422. Antibody details are provided in Table 1.

**Table 1:** Immunohistochemical antibody information

Antibody	Clone	Vendor	Dilution & pretreatment	Detection system
SARS-CoV-N	Polyclonal	Antibodies online	1:1000 &	Ultra view red Ventana Benchmark Ultra
CD68	PG-M1	Dako	1:100 & VA	Ultra View Red
				Ventana Benchmark Ultra

Tissue RNA extraction for SARS-CoV-2 testing was performed on the Maxwell 16 MDx instrument by Promega using the Maxwell 16 LEV RNA FFPE Purification Kit according to the manufacturer's protocol. In each case, four 10 µm slices of full thickness placental parenchyma were used. For the analyses of SARS-CoV-2 in the fetal parenchyma, two 10 µm slices of every organ examined in the autopsy were pooled in one tube with subsequent RNA dilution. For SARS-CoV-2 detection, a real time reverse transcriptase (RT)-PCR assay with specific amplification of the ORF1ab and N conserved regions of SARS-CoV-2 was used (AmoyDx® Novel Coronavirus (2019-nCoV) Detection Kit, Amoy Diagnostics Co., Ltd.). Lung tissue from a non-related SARS-CoV-2-positive autopsy served as a positive control.

Presence of SARS-CoV-2 RNA in nasopharyngeal swabs was assessed by real time RT-PCR targeting the viral RdRp and N-genes (Alinity m SARS-CoV-2 assay, Abbott Molecular, Des Plaines, IL, USA) following the manufacturer’s instructions. Viral whole-genome sequences were obtained using the EasySeq RC-PCR SARS CoV-2 kit (NimaGen B.V., Nijmegen, The Netherlands) and a NextSeq sequencing system (Illumina, San Diego, CA, USA). Viral sequences were aligned and evaluated using Geneious Prime software (Biomatters, Auckland, New Zealand). Viral lineages were assessed by the Pangolin COVID-19 Lineage Assigner.

## Results

Case 1: A 30-year-old II-gravida I-para was admitted to the hospital with abdominal pain and intrauterine death at 27 + 3 weeks of gestation. There were no clinical signs consistent with COVID-19. However, presence of SARS-CoV-2 RNA in the mother’s nasopharyngeal swab was detected by RT-qPCR (cycle threshold 25) on the day of admission. Subsequent viral whole genome sequencing and phylogenetic analysis identified the SARS-CoV-2 Alpha variant (Pangolin lineage B.1.1.7) in the swab.

The female fetus of 730 g (20th percentile) and 24.4 cm crown-rump length (< 10th percentile) showed no intrauterine growth restriction. No malformations nor inflammatory changes were detected on autopsy.

Immunohistochemical staining of all fetal organs and RT-PCR testing of the fetal tissue was negative for SARS-CoV-2 (Fig. 3a,b).

**Fig. 1:**
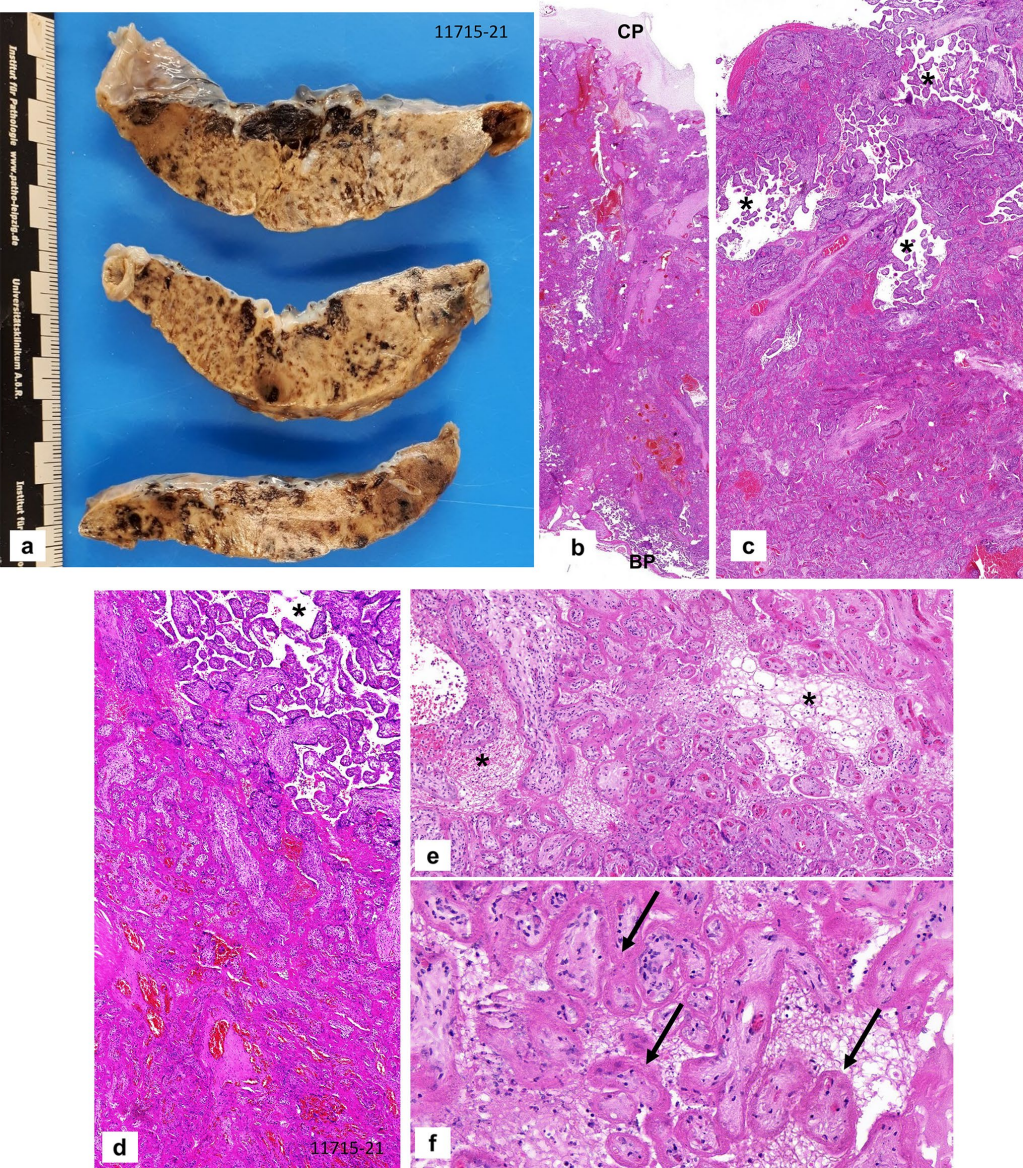

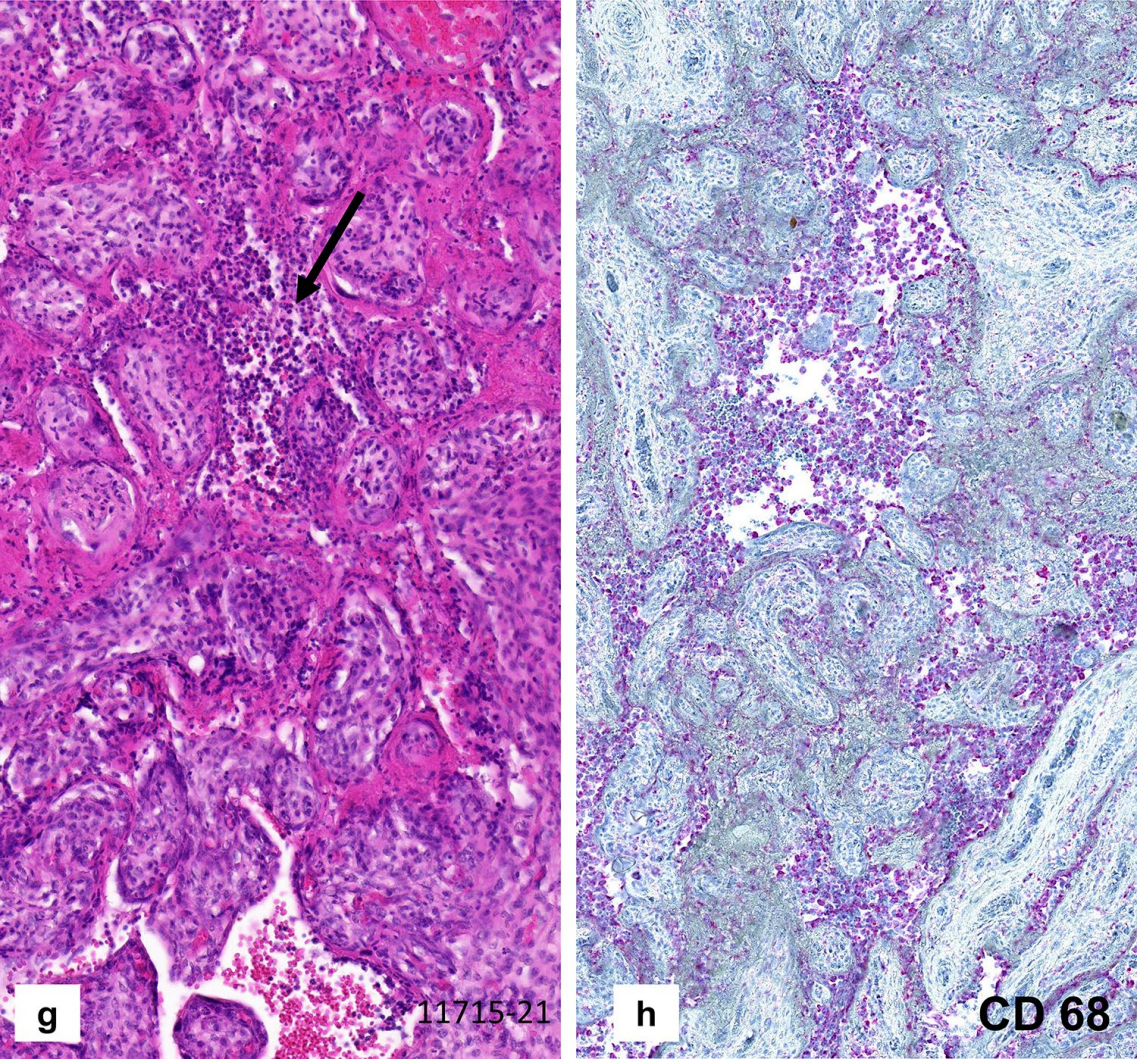
Case 1: Placenta from intrauterine demise at 27 + 3 weeks of gestation. **a** Cutting surface of the stiffed placenta showing a pale net-like and pearly appearance involving more than 95% of the whole placenta. **b** The whole thickness of the placenta is involved by extensive perivillous fibrin deposits involving the placental tissue from the chorionic plate (CP) to the basal plate (BP). **c** Occasional groups of preserved chorionic villi with open intervillous space (*) within the placental tissue. **d** Higher magnification from **c** highlighting the obliteration of the intervillous space by extensive (clotted) perivillous fibrin deposits close to a small area of open intervillous space (*). **e** Fresh obliteration of the intervillous space with net-like appearing fibrin (*). **f** Extensive necroses of the villous trophoblast with pale red staining without visible trophoblastic nuclei (arrows). **g** Focal area of chronic histiocytic intervillositis (arrow) is present together with intervillous fribrin deposits and early trophoblastic necroses. **h** Highlighting the histiocytic infiltration by CD68 immunohistochemistry

**Fig. 2:**
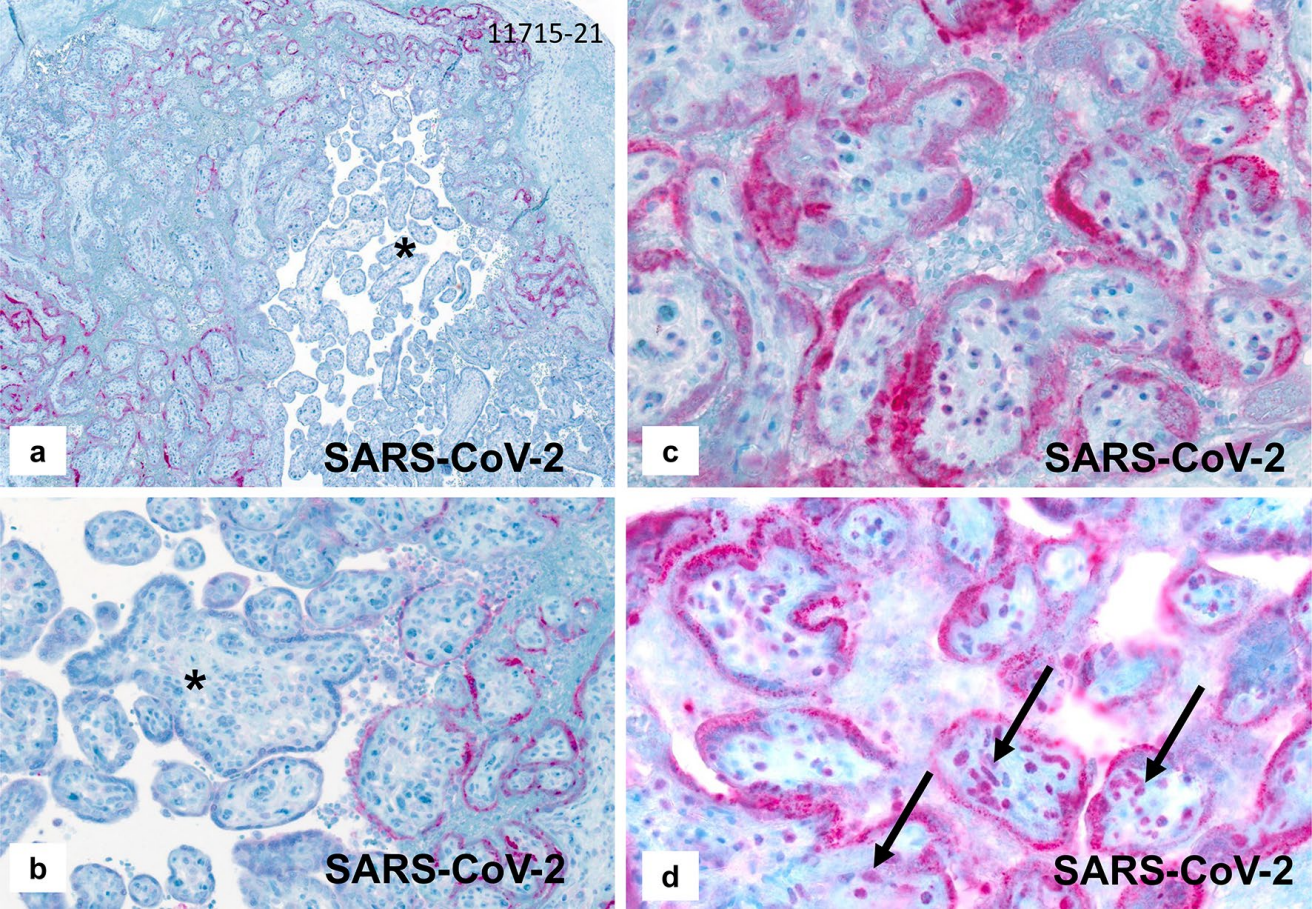
Case 1: Immunostaining for SARS-CoV-2. **a**, **b** Positive staining for SARS-CoV-2 is restricted to the necrotic villous trophoblast whereas preserved villi with open intervillous space (*) are negative. **c** Strong and extensive staining for SARS-CoV-2 within the villous trophoblastic cells. **d** In addition to the villous trophoblastic cells, positive staining is also present in several villous stromal cells (arrows)

**Fig. 3:**
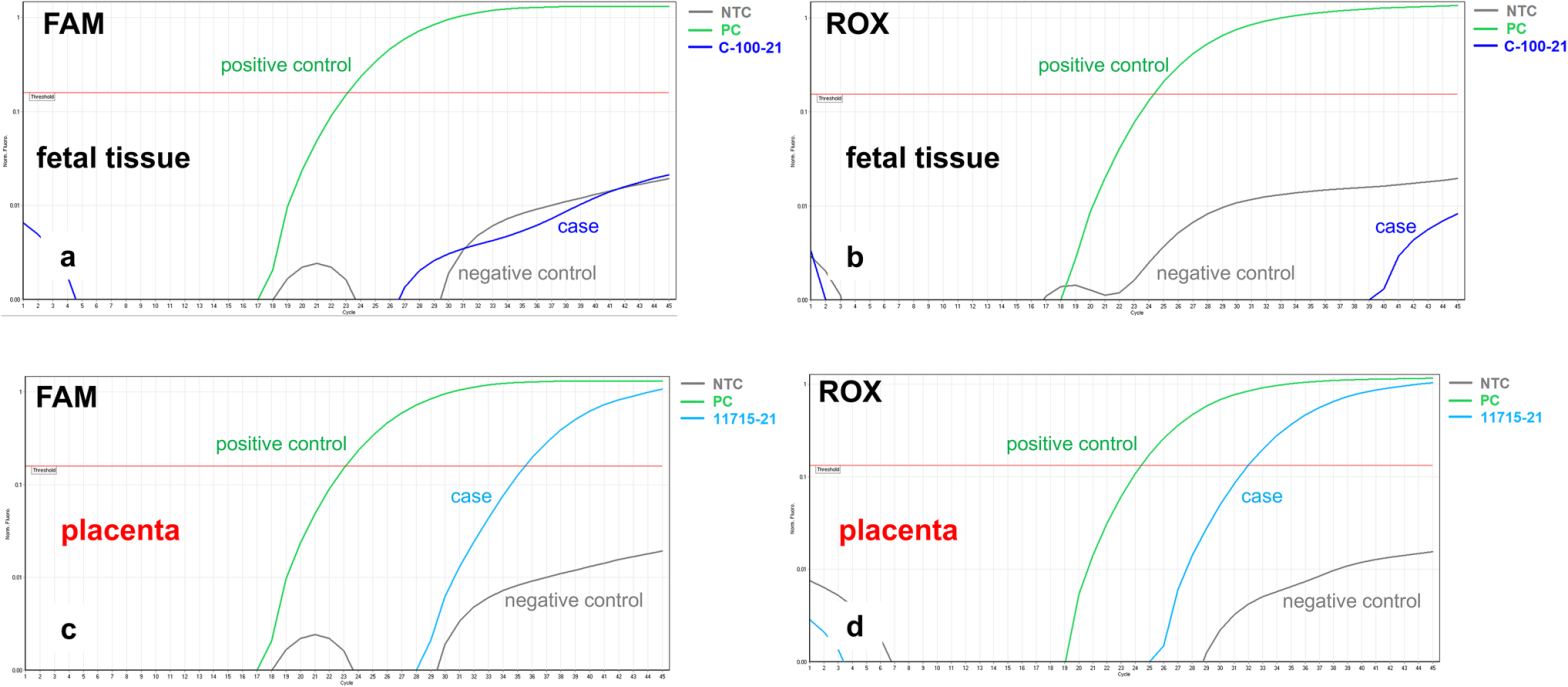
Case 1: RT-PCR results for SARS-CoV-2. **a**, **b** SARS-CoV-2-specific RT-PCR FAM-/ROX fluorescence signals were negative for the fetal tissue. **c**, **d** SARS-CoV-2-specific RT-PCR amplification plots showing positive FAM-/ROX fluorescence signals within the placental tissue (please see text for details)

The placenta weighed 159 g (10th percentile), measured 13 × 10 × 1.8 cm and demonstrated an increased consistency and a pale, stiff, net-like cutting surface (Fig. 1a) over more than 95% of the whole organ. Histologically, massive perivillous fibrin deposits were seen comprising > 90% of the parenchyma (Fig. 1b–e), associated with extensive trophoblastic necroses (Fig. 1f). There was no villous dysmaturity. However, chronic histiocytic intervillositis was seen very focally and the inflammatory cells were positive for CD68 in immunohistochemistry (Fig. 1g, h). The villous trophoblast showed strong immunopositivity for SARS-CoV-2 with necrotic alterations (Fig. 2a–c). Very focally, there were villous stromal cells with positive staining (Fig. 2d). Immunohistochemical staining was negative within the chorionic and basal plate as well as the fetal membranes and the umbilical cord. RT-PCR confirmed the presence of SARS-CoV-2 RNA in the placental tissue, showing cycle thresholds of 32 and 35, respectively (Fig. 3c, d).

**Fig. 4:**
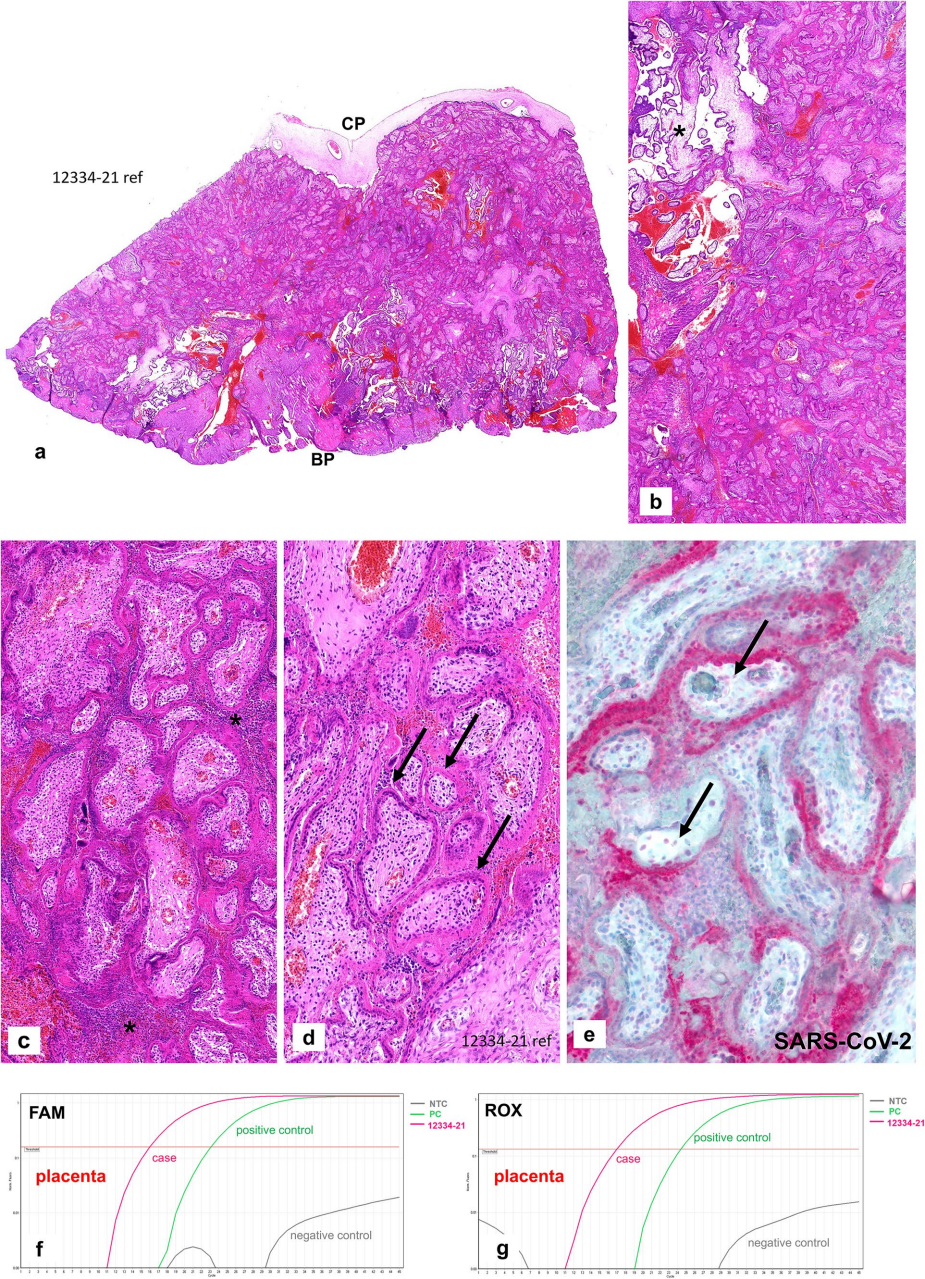
Case 2: Placenta from intrauterine demise 15 + 2 weeks of gestation. **a** Extensive perivillous fibrin deposits involving the placental tissue from the chorionic plate (CP) until basal plate (BP). **b** Very focal area of preserved chorionic villi with open intervillous space (*) within the placental tissue. **c** Focal areas of histiocytic intervillositis (*) within intervillous fribrin deposits. **d** Fresh necroses of the villous trophoblast with a pale red appearing of the villous trophoblast, some trophoblastic nuclei are still preserved (please compare to Fig. 1e, f). **e** Extensive and strong staining of the villous trophoblastic cells for SARS-CoV-2, some villous stromal cells with weak staining (arrows). **f**, **g** SARS-CoV-2-specific RT-PCR amplification plots with positive FAM-/ROX fluorescence signals within the placental tissue. Compared to Figs. 3c and d, there is a higher viral load in case 2, indicated by early and strong increase of the plotting curves which is stronger than the positive control curve

Case 2: A 39-year-old II-gravida 0-para was admitted to the hospital at 15 + 2 weeks of gestation with an intrauterine demise. She had developed a febrile cough ten days prior to the hospital admission. A naso-pharyngeal swab was positive for COVID-19, detecting the Alpha variant on RT-PCR. The demised male fetus weighed 40 g (< 10th percentile) and had a crown-rump-length of 9.5 cm (< 10th percentile) consistent with an intrauterine growth restriction. No malformations or inflammatory changes were seen. Immunohistochemical analyses of all fetal organs were negative for SARS-CoV-2. The placental disc showed a partial pale cutting surface with stiffness of the parenchyma, occupying approximately 50% of the placental tissue. Microscopically, there were massive perivillous fibrin deposits occupying up to 90% of the placental tissue from the chorionic to the basal plate (Fig. 4a, b). The placental villi showed no dysmaturity but very focal histiocytic chronic intervillositis (Fig. 4c). The histiocytes were positive for CD68 on immunohistochemistry (not shown). There were extensive necroses of the villous trophoblastic cells (Fig. 4d). The villous trophoblast showed strong and diffuse staining for SARS-CoV-2 with weak positivity of some villous stromal cells (Fig. 4e). Only trophoblastic villi surrounded by intervillous fibrin stained positive for SARS-CoV-2. The chorionic plate, umbilical cord and fetal membranes were negative for SARS-CoV-2. RT-PCR analysis of the placental tissue showed high viral loads (cycle threshold 16 and 17, respectively) (Fig. 4f, g). No fetal tissue was available for RT-PCR testing in this case.

## Discussion

There is an increasing number of studies examining placental morphology in mothers diagnosed with COVID-19. Some studies examined placentas from COVID-19-negative and COVID-19-positive mothers [e.g. 19]. However, the majority of reports as well as a recent review [12] evaluate mothers with symptomatic SARS-CoV-2 infections [13–15, 22, 24] with various fetal outcomes [13–16]. These results suggest that placental findings must be strictly interpreted in the context of fetal outcome and maternal symptoms.

The vast majority of studies did not include the macroscopic findings of the placentas of SARS-CoV-2-infected women [9, 13–16]. Schoenemakers et al. [23] reported a “dense and stiff placenta with pale trabecula in a lattice network”. Hosier et al. [24] described a focal placental infarct. Rebutini et al. [14] reported some infarcted areas in 3/18 (16.7%) cases covering < 5% of the placental tissue from live born infants in contrast to infarcted areas of 30–40% in a case with intrauterine death at 28 + 3 weeks of gestation. Morton et al. [25] published a pearly, firm, netlike cutting surface in a case of intrauterine death. Garrido-Pontnou et al. [22] described stiff placental disks with an increased consistency as striking features in association with fetal demise consistent with one case of Watkins et al. [49]. In the present cases, one placenta demonstrated a dense and whitish cutting surface with a markedly increased consistency incorporating more than 90% of the whole organ (see Fig. 1a). In the other case there was a pale, stiff appearance of about 50% of the tissue.

A systematic analysis of the available literature up to November 30th, 2020 revealed that the majority of studies evaluated placentas from SARS-CoV-2 positive mothers only while controls for comparison were rarely used [12, 14, 17, 47].

One of the leading finding in placental histology in SARS-CoV-2-infected mothers, regardless of fetal outcome, is evidence of maternal vascular malperfusion [9, 12, 14, 15, 49].

Mixed chronic, histiocytic-predominant intervillositis [13, 19, 47], as signs of inflammatory changes may occur in SARS-CoV-2 infection [16, 18, 19]. However, this feature may be rare [9, 12, 14] and might be associated with a severe maternal COVID-19 course [14]. Schwartz et al. [10] as well as Watkins et al. [49] suggested that histiocytic intervillositis may be more predominant in SARS-CoV-2-infected mothers with live born infants than in cases with intrauterine demise. Although villous dysmaturity has been reported in women affected by SARS-CoV-2 [12, 15] its frequency is not different to matched controls [14].

In an analysis of placentas with pathologists blinded to the patients’ COVID-19 status, no differences were seen for the presence of villitis, chorioamnitis, intervillositis and increased numbers of decidual lymphocytes [17, 20, 21].

Another common feature seen in placentas from SARS-CoV-2-affected mothers is an increase in intervillous fribrin deposition [9, 12, 13, 22, 50]. Compared to controls, significantly increased intervillous fibrin deposits were present in SARS-CoV-19 placentas (9/27 versus none; *p* = 0.036; [20]. Smithgall et al. [21] reported significantly more subchorial intervillous fibrin deposits in SARS-CoV-2-positive cases compared to negative ones (9/51 versus 0/24; *p* = 0.026).

Lu-Culligan et al. [20] showed no association between intervillous fibrin deposits and the presence of COVID-19 symptoms, maternal and fetal co-morbidities, mode of delivery or BMI. In contrast, Smithgall et al. [21] noted that placentas from symptomatic SARS-CoV-2-positive mothers showed more subchorial fibrin deposits than asymptomatic ones (18% versus 8%; *p* = 0.07).

In addition, Shanes et al. [15] reported increased intervillous fibrin in 46.7% of the placentas of live born infants within the 3rd trimester. A morphometric analysis showed more accentuated placental fibrin deposits in SARS-CoV-2-infected mother, compared to controls (*p* = 0.08; [14]).

Intervillous fibrin deposits have been reported to cause fetal distress [13, 14, 16, 22, 23]. Within an analysis of published cases Sharps et al. [12] reported that 10.8% of the live born infants of COVID-19 infected mothers were small for gestational age (SGA) or were recorded as < 2.500 g at birth (with no data provided if the birthweights were appropriate for gestational age) when increased intervillous fibrin deposits were seen. Within a systematic review Wei et al. [30] showed that COVID-19 was associated with an odds ratio of 2.11 [95% CI (1.14–3.90)] for intrauterine death. That finding was supported by a recent analysis of DeSisto et al. [48]. Interestingly, the relative risk for intrauterine death was 1.47 [95% CI = 1.27–1.71] in the pre-Delta-period compared to an increased risk in the Delta-period [RR = 4.04 (95% CI = 3.28–4.97); 48]. Both women of the present report were tested positive for the Delta-variant of SARS-CoV-2.

Very extensive intervillous fibrin has been reported in placentas from the second trimester [12], especially in those associated with fetal miscarriage including our present cases [13, 16, 18, 22, 24, 25].

Jak et al. [6] reported extensive intervillous fibrin deposits in a placenta at 39 weeks of gestation, resulting in an intrauterine growth restriction of the fetus with a postnatal death. In the report of Poisson and Perone [26], approximately 75% of the intervillous space was occupied by fibrin deposits, accompanied by intrauterine fetal death at 35 weeks of gestation. Hosier et al. [24] described diffuse perivillous fibrin in the placenta of a 22 week fetal demise. Marton et al. [25] reported massive intervillous fibrin deposits occupying up to 90% of the intervillous space in a 25 + 5 week miscarriage. Schwartz et al. [13] described extensive perivillous fibrin deposits in all their cases with intrauterine death.

It is interesting to note that the vast majority of cases including the present case 1 with massive intervillous fibrin deposits and intrauterine death show no intrauterine growth restriction [12, 13, 24–26]. The absence of fetal growth restriction in association with massive intervillous fibrin deposits suggests a rapid onset of the fibrin deposition.

Intervillous fibrin has not only been seen in placentas from COVID-19-positive women. It is known as a histopathological feature that increases with decreased uterine perfusion, increased maternal coagulability and decreased thrombolytic function of the trophoblastic cells [27, 28]. Increased intervillous fibrin deposits have been reported in intrauterine death in several conditions [28, 29] prior to SARS-CoV-2 pandemic. In the present cases, extensive intervillous fibrin deposits were associated with intrauterine death. This feature was reported in almost all cases with fetal demise in SARS-CoV-2-positive mothers [13, 16, 18, 24, 26, 30]. As a conclusion, the obstruction of the intervillous space by fibrin deposits results in fatal fetal hypoxia, responsible for intrauterine death.

However, the reasons for the development of intervillous fibrin deposits remains unclear [50].

Aside from respiratory symptoms, SARS-CoV-2 has been reported to cause severe coagulation disorders in affected patients [2, 31]. This finding has been explained by several hypotheses, including a cytokine or microparticle storm, complement factor activation and direct viral interaction with receptors [1, 32]. Based on the current COVID-related knowledge, the coagulopathies in patients with SARS-CoV-2 infections are attributed to a combination of different factors rather than one single mechanism [1, 2, 32]. Regarding the fibrin deposits in the intervillous space of the placentas from SASRS-CoV-2-positive mothers with fetal distress, there may be a decrease in local fibrinolysis similar to what is seen in preeclampsia [33, 34]. Perivillous fibrin deposition may be triggered by an activation of immune cells and circulating pro-inflammatory cytokines [20] and potentially microparticles [32] from the mother as well as by the hypoxic alteration of the villous trophoblast [33, 35, 36]. Therefore, intervillous fibrin deposition may also be a multifactorial pathogenetic process in placentas affected by SARS-CoV-2 [13, 20, 33]. In contrast, placentas from mothers without maternal vascular malperfusion may demonstrate unspecific histiocytic intervillositis with marginal fibrin deposits not affecting the fetus. Overall, inflammatory changes in placentas from SARS-CoV-2-postive mothers may not differ from controls [14, 15]. In both present cases with intrauterine failure, very limited histiocytic intervillositis was seen (Figs. 1g, h, 4c).

Consistent with the cases described in this publication (see Figs. 1e, f, g, 4d), necroses of the villous trophoblast have previously been reported [13, 22, 50, 51]. In cases with necrotic trophoblastic cells, SARS-CoV-2 was detected within the trophoblastic epithelium by immunohistochemistry (see Figs. 2, 4e for the present cases) and/or in situ hybridization [9, 13, 19, 22, 24, 51]. The antibody used for evaluation described in this manuscript is directed against the SARS-CoV-2 nucleocapsid protein. This indicates a specific staining for SARS-CoV-2-virus which has been confirmed by additional RNA-analyses in the present cases and other studies [13, 19, 21]. Interestingly, positive immunohistochemical staining for SARS-CoV-2 was restricted to chorionic villi surrounded by intervillous fibrin showing trophoblast cell necroses (Fig. 2 a, b). This feature has previously neither been mentioned nor illustrated by others [9, 13, 20, 24, 25]. As shown in Fig. 2d in the paper of Garrido-Pontnou et al. [22] SARS-CoV-2 villous positivity was restricted to villi embedded in dense perivillous fibrin. It remains unclear if the trophoblastic damage is caused by a direct viral effect or is secondary to inflammatory or ischemic injury [14, 22]. Within the authors’ opinion, the latter pathogenetic mechanism is more reliable since trophoblastic necroses are also seen in placentas of women with hypertensive disorders known to be associated with intervillous hypoxia [27, 28, 37]. Whichever mechanism is involved, damaging the protective function of the villous trophoblast can facilitate villous stromal infection with vertical transmission of SARS-CoV-2 to the fetus. As can be seen in Figs. 2d, 4e, SARS-CoV-2 can be immunohistochemically detected in villous stromal cells.

Villous trophoblastic cells represent the cellular barrier between the maternal and fetal vascularisation. They are in direct contact with the maternal blood and are therefore permissive for viral infection, e.g. in SARS-CoV-2 [38]. In SARS-CoV-2-infected placentas with trophoblastic necroses, that barrier is disturbed and the trophoblast is permeable for the virus which can then infect the villous stromal cells.

Garrido-Pontnou et al. [22] proposed that the diffuse trophoblastic damage seen in SARS-CoV-2-infected placentas [9, 13, 19, 24] and characterised by trophoblastic necroses, variable number of histiocytic intervillositis, perivillous fibrin deposition and intervillous space collapse may represent the placental counterpart of the diffuse alveolar damage of the lung in COVID-19.

Sharps et al. [12] reported in a literature review that the studies examining the tissue for SARS-CoV-2 by immunohistochemistry and/or in situ-hybridization described a 2% positivity of the neonates and 21% of the placental tissue for the virus. Several studies reported positive immunostaining for SARS-CoV-2 within the syncytiotrophoblast [13, 23, 24, 47, 50]. In the analysis of Rebutini et al. [14], none of the eleven placentas in live born infants tested for SARS-CoV-2 RNA was positive. The only case positive within the placenta resulted in an intrauterine demise. Gao et al. [9] reported negative results for the detection of SARS-CoV-2 by immunohistochemistry and FISH-analysis in the placentas of live born fetuses from the 3rd trimester. Within a total of 198 SARS-CoV-2-positive mothers, only nine placentas (4.5%) showed SARS-CoV-2-positivity within the villous syncytiotrophoblast either by immunohistochemistry and/or RT-PCR, suggesting placental infection within the analyzed cases of live born infants [9].

Schoenemakers et al. [23] reported fetal distress and neonatal multi-organ failure in an asymptomatic mother. For example, in a case of a 16 week intrauterine demise, the mother was asymptomatic [15]. Both mothers of the present cases were non- or oligosymptomatic for COVID-19.

The majority of pregnant women tested positive for SARS-CoV-2 by RT-PCR on nasopharyngeal swabs within a prospective study were asymptomatic (75.2% (103/137); [8]. This is consistent with other studies [14–16, 39]. In the second case described in this publication, the intrauterine death occurred at day 10 of the maternal COVID-19 infection.

The incidence of intrauterine fetal demise was significantly higher in SARS-CoV-2-infected mothers compared to uninfected ones, with a reported relative risk of 4.7 [(1.4–15.9); *p* = 0.0057] [8]. All stillbirths occurred in asymptomatic (85.7%) or oligosymptomatic (14.3%) mothers. Four of five women reported by Richtmann et al. [16] were non- or oligosymptomatic.

Intrauterine deaths occurred over a wide range of time in women with COVID-19, ranging from day one to day 22 of the COVID infection [13, 15, 16, 24].

Transplacental (vertical) transmission of SARS-CoV-2 is not a prerequisite for an intrauterine death, and the majority of pregnancies with fetal demise are more likely to be caused by placental failure [14], [12–14, 16, 24, 30] rather than the viral infection of the fetus itself.

Vertical transmission has been reported for several maternal virus infections, either via direct viral transmission or using placental Hofbauer cells as carriers [40, 41, 51]. In contrast, vertical transmission in SARS-CoV-2 remains controversial [5, 8, 10, 42], including the pathogenetic route of the virus [10, 20, 23]. As in the present cases, SARS-CoV-2 can be immunohistochemically detected within the trophoblastic cells, lining the placental villi at the feto-maternal interface (Figs. 2, 4e); [13, 19, 23, 24, 47, 50]. In contrast to other viral infections [40], SARS-CoV-2 does not appear to productively infect placental Hofbauer cells in vitro or in vivo [20]. In contrast to Zika-virus infections [51], there was no increase of intravillous Hofbauer cells in COVID-19-positive mothers [9, 47], not even when compared to controls [14].

Rarely, SARS-CoV-2- antigen expression has been reported in villous Hofbauer cells [19, 47] but the finding was not illustrated in the paper. Dong et al. [10] described positive immunostaining for SARS-CoV-2 within Hofbauer cells but also did not illustrate this finding. In contrast, a very recent paper by Schwartz et al. [47] reported positive SARS-CoV-2 immunostaining within Hofbauer cells in 4 out of 22 cases (18.2%). SARS-CoV-2-positivity within Hofbauer cells was associated with trophoblast necroses of the chorionic villi in all their cases. However, there was no correlation with fetal outcome. While the number of cases examined was relatively small, Schwartz et al. [47] did not demonstrate an association between the viral staining of Hofbauer cells and a greater probability of maternal–fetal transmission or poor outcome.

Dong et al. [10] showed SARS-CoV-2-positive villous stromal cells in Figs. 2A, C and D of their publication. In the present cases, SARS-CoV-2-positive villous stromal cells were only seen in chorionic villi with necrotic trophoblastic cells on their surface. Immunohistochemical staining for fetal membranes, umbilical cords and all fetal tissue was negative in both of our cases. The fetal tissue tested by RT-PCR from one of our cases was negative for SARS-CoV-2 (Fig. 3a,b).

Extrapolating from the data in the literature it may be assumed that mother-to-fetus SARS-CoV-2 transmission does occur, but it is infrequent [12, 14, 19]. Two recent systematic reviews reported a vertical transmission rate of 5.7% [43] and 3.3% [44].

The exact mechanism of intrauterine SARS-CoV-2- transmission is not fully understood. However, two hypotheses may be abstracted from the literature.

The spike (S) protein of SARS-CoV-2 binds to its cell adhesion and soluble receptor ACE-2 [45], which is also present in the placental trophoblast [10, 20].

Lu-Culligan et al. [20] described a significantly stronger and more diffuse expression of the ACE-2 protein within the cyto- and syncytiotrophoblastic cells in the placental tissue between 7 and 15 weeks of gestation, with a significant decrease after 21 weeks. Dong et al. [10] reported low ACE-2 staining in placental tissue in the third trimester. These results suggest that the end of the first and the beginning of the second trimester may be a more vulnerable period for the acquisition of placental SARS-CoV-2 infections compared to the third trimester of pregnancy [20].

Second, the virus might pass the placental barrier because of damages caused by intervillous hypoxia due to vascular and hemodynamic disturbances in the mother [13, 38, 46]. As mentioned above, necroses of the syncytiotrophoblast have been reported by several authors [12, 13, 16, 24, 26, 47, 50] and were also seen in the present cases (Figs. 1c, e, 4d).

The morphologic findings in placentas from SARS-CoV-2-positive mothers with and without a fetal demise are summarised in Table 2. The reporting and collecting of data of a SARS-CoV-2 infection in pregnancy is important to offer patients optimal treatment and to help health professionals develop appropriate management protocols for these women [6]. In cases with an adverse pregnancy outcome, a histopathological examination of the placental and fetal tissue by immunohistochemical and molecular techniques to verify SARS-CoV-2 is mandatory to determine the pathogenesis and understand the functionality of the disease.

**Table 2:** Clinicopathologic features in placentas from SARS-CoV-2 positive mothers with and without fetal demise [^6,12,13,20,22,47,49,50^, present cases]

• Overall, placental infection by SARS-CoV-2 is seen in < 10% of SARS-CoV-2-positive mothers,
• Morphologic pattern of placental infection include:
Chronic histiocytic predominant intervillositis (mostly seen in cases with live born infants),
Trophoblastic necrosis,
Intervillous space collaps and
Variable amount of perivillous fibrin deposition,
• Placentas from SARS-CoV-2-positive mothers without fetal distress may represent no or only little intervillous fibrin deposits and/or histiocytic intervillositis,
• In pregnancies affected by fetal distress, intrauterine growth restriction or even intrauterine death, increased intervillous fibrin deposits are present, mostly in placentas from the second trimester,
• Reported increased perivillous fibrin deposition is very rarely associated with fetal growth restriction compared to intrauterine death, suggesting rapid onset of intervillous fibrin deposition resulting in acute intervillous hypoxia
• Intervillous fibrin deposits represent a multifactorial pathogenetic process caused by a combination of maternal hypercoagulation process resulting in intervillous hypoxic conditions and perhaps a direct damage of the trophoblastic cells on the villous surface by cytokine/microparticle storm and cytokine activation
•may be caused by increased trophoblastic ACE-2-receptor expression, 1st and 2nd trimester represent the most vulnerable time period for placental SARS-CoV-2 infection,
• Although the detection of SARS-CoV-2 within the placental tissue has been reported using in situ-techniques or immunohistochemistry, that feature is not as common in SARS-CoV-2-positive mothers,
• Adverse clinical outcome may also be seen in asymptomatic mothers or those with subclinical COVID-19
